# Functions of Danggui Buxue Tang, a Chinese Herbal Decoction Containing Astragali Radix and Angelicae Sinensis Radix, in Uterus and Liver are Both Estrogen Receptor-Dependent and -Independent

**DOI:** 10.1155/2014/438531

**Published:** 2014-08-19

**Authors:** Oliver Zierau, Ken Y. Z. Zheng, Anja Papke, Tina T. X. Dong, Karl W. K. Tsim, Günter Vollmer

**Affiliations:** ^1^Institute for Zoology, Molecular Cell Physiology and Endocrinology, Technical University Dresden, 01062 Dresden, Germany; ^2^Division of Life Science and Center for Chinese Medicine, The Hong Kong University of Science and Technology, Clear Water Bay Road, Hong Kong; ^3^Department of Biology, Hanshan Normal University, Chaozhou, Guangdong 521041, China

## Abstract

Danggui Buxue Tang (DBT), a herbal decoction containing Astragali Radix (AR) and Angelicae Sinensis Radix (ASR), has been used in treating menopausal irregularity in women for more than 800 years in China. Pharmacological results showed that DBT exhibited significant estrogenic properties *in vitro*, which therefore suggested that DBT could activate the nuclear estrogen receptors. Here, we assessed the estrogenic properties of DBT in an ovariectomized *in vivo* rat model: DBT was applied to the ovariectomized rats for 3 days. The application of DBT did not alter the weight of uterus and liver, as well as the transcript expression of the proliferation markers including the estrogen receptors *α* and *β*. However, DBT stimulated the transcript expression of the estrogen responsive genes. In addition, the inductive role of DBT on the expression of members of the aryl hydrocarbon receptor family in uterus and liver of ovariectomized rats was confirmed. These responses of DBT however were clearly distinct from the response pattern detectable here for 17*β*-estradiol. Therefore, DBT exhibited weak, but significant, estrogenic properties *in vivo*; however, some of its activities were independent of the estrogen receptor. Thus, DBT could be an exciting Chinese herbal decoction for an alternative treatment of hormone replacement therapy for women in menopause without subsequent estrogenic side effects.

## 1. Introduction

Traditional Chinese medicines (TCMs) have been used as medicines or health food supplements in China for over thousand years. Historically, TCMs are prepared as decoctions by a unique methodology with specific combinations of different herbs as a formula. Among thousands of herbal formulae, Danggui Buxue Tang (DBT) is a simple herbal decoction that is composed of two herbs. DBT was first described in* Neiwaishang Bianhuo Lun* by* Li Dongyuan* in China in AD 1247.* Li *described that the DBT formula should include 10* qian* of Astragali Radix (AR), roots of* Astragalus membranaceus* (Fisch.) Bunge or* Astragalus membranaceus* (Fisch.) Bungevar.* mongholicus *(Bunge) P.K. Hsiao, and two* qian* of Angelicae Sinensis Radix (ASR), roots of* Angelica sinensis* (Oliv.) Diels. One* qian* equals about 3 grams. DBT is prescribed for women in China as a remedy for menopausal symptoms, which improves their health by raising the “*Qi*” (vital energy) and nourishing the “*blood*” (body circulation).

Women in menopause suffer from hot flushes, sweating, anxiety, and mood swings, as well as from an increased risk for many health problems, such as loss of bone density mass (osteoporosis) and cardiovascular disease. These problems are largely due to the deficiency of ovarian hormones, especially estrogens [1]. Hormone replacement therapy (HRT) had been used to alleviate menopausal symptoms for many years, but this treatment was associated with side effects, that is, an increased risk of breast cancer, heart attacks, and strokes [2]. In view of these clinical risks, extensive efforts have been devoted to searching or developing new drugs that would yield the benefits of hormone therapy but with minimal risk [3]. TCM, containing thousands of medicinal herbs, is a promising resource that could provide a perfect solution [4]. Indeed, some of the herbal products showed a pronounced efficacy for menopausal symptoms and as a consequence were widely used by women to relieve their menopausal symptoms, for example, DBT [5–8].* In vitro* experiments with mammary gland [6] and bone [9] derived cells suggested that at least some of the effectiveness of DBT is mediated through estrogen receptor- (ER-) dependent mechanisms. In order to support these observations* in vivo*, DBT was tested in the ovariectomized Wistar rat model for potential estrogenic properties. Since DBT is a decoction that traditionally is consumed as a tea, animals were supplemented by DBT through the drinking water. Taking this approach, we performed a three-day uterotrophic assay for revealing responses relevant to contribute to the elucidation of the molecular mechanisms of action of DBT, including organ weights and regulation of gene expression.

## 2. Materials and Methods

### 2.1. Plant Materials and DBT Preparation

Three-year-old AR deriving from the roots of* A. membranaceus *var.* mongholicus* was collected from Shanxi province [10], and 2-year-old ASR of* A. sinensis* was from Minxian in Gansu province [11]. These plant materials had been morphologically authenticated by Dr. Tina Dong, during the field collection. The corresponding vouchers as forms of whole plants, voucher # 02-9-1 for ASR and voucher # 02-10-4 for AR, were deposited in the Center for Chinese Medicine, The Hong Kong University of Science and Technology. 17*β*-Estradiol (E_2_) was obtained from Sigma-Aldrich (Deisenhofen, Germany). 250 g of sliced AR and 50 g of sliced ASR were mixed (the ratio is 5 : 1) and then boiled in 2,400 mL (w : v = 1 : 8) of water for 2 hours, and then the decoction was filtered. The residues were boiled in 1,800 mL (w : v = 1 : 6) water for 1 hour. The combined extracts were dried under vacuum and stored at −20°C. This extraction, following the ancient preparation, was shown to be the best extracting condition [12]. Two chemical markers in AR (calycosin and formononetin) and two others in ASR (ferulic acid and ligustilide) were used to standardize the chemical property of DBT. The standardized DBT should contain no less than 0.186 mg calycosin, 0.155 mg formononetin, 0.351 mg ferulic acid, and 0.204 mg ligustilide per one g dried weight of DBT, as reported previously [5, 6, 8].

### 2.2. Animals

Juvenile female Wistar rats (130 ± 15 g) were obtained from Harlan Winkelmann (Borchen, Germany) and were maintained under controlled conditions of temperature (20°C ± 1, relative humidity 50–80%) and illumination (12 hours light, 12 hours dark). All rats had free access to standard rat diet (SSniff R10-Diet, SSniff GmbH, Soest, Germany) and water. All animal husbandry and handling conditions were according to the Institutional Animal Care and Use Committee Guidelines in Germany.

### 2.3. Uterotrophic Assay

**Figure 1:**
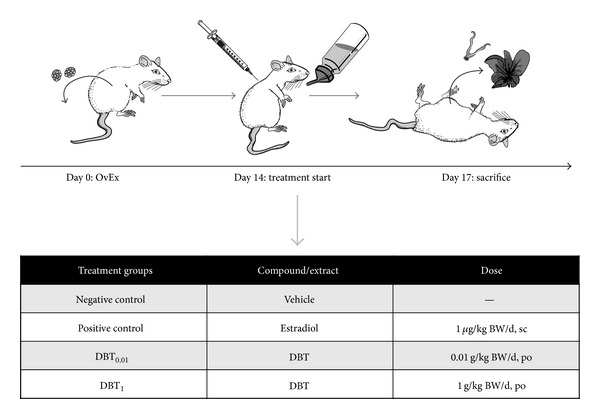
Experimental design. The figure summarizes the experimental design of the study. It schematically shows the workflow, as well as the summary of the treatment groups included. bw: body weight; DBT: Danggui Buxue Tang; DBT_01_ and DBT_1_: DBT at 0.01 and 1 g/kg bw/d.

The estrogenicity was tested in the 3-day assay in ovariectomized rats according to the OECD guideline 440 [13]. The experimental procedures are schematically summarized in Figure 1. Briefly, following ovariectomy and 14 days of endogenous hormonal decline, the animals were treated for 3 days. The animals were randomly allocated to the treatment with herbal extracts, positive control, or vehicle groups (*n* = 6). DBT was administered orally at the doses of 0.01 g/kg BW/d or 1 g/kg BW/d BW and E2 (1 *μ*g/kg BW/d; subcutaneous injection), which served as a positive control. Animals were sacrificed by decapitation after light anaesthesia with CO_2_ inhalation. The wet weights of uterus and livers were determined. Uteri and livers were frozen in liquid nitrogen for the RNA preparation.

### 2.4. Total RNA Preparation and Reverse Transcription

The total cytoplasmic RNA was extracted from the rat uteri by the standard TRIzol method (Invitrogen, Grand Island, NY). DNA residues were enzymatically eliminated by digestion (Deoxyribonuclease I, Ambion, Foster City, CA), and the removal was checked by PCR. Superscript II Reverse Transcriptase (Invitrogen) and Oligo (dT) 12–18 were used for the first-strand cDNA synthesis.

### 2.5. Quantitative Real-Time PCR

Quantitative real-time PCR was carried out by Platinum Taq DNA polymerase (Invitrogen) using the iCycler thermal cycler with iQ real-time detection system. The reactions were run three times in triplicate. After vortexing, 50 *μ*L aliquots of the mix were pipetted in each well of the 96-well thin-wall PCR plate (Bio-Rad, Hercules, CA). PCR reactions consisted of a first denaturing cycle at 95°C for 3 min, followed by 50 cycles of 10 s at 95°C, 15 s at 60°C, and 30 s at 72°C. Fluorescence was quantified at the end of the 60°C annealing step and product identity was confirmed by a melting curve analysis (60–95°C). Primer sequences and amplicon sizes are summarized in the Supplementary Table 1 in Supplementary Material available online at http://dx.doi.org/10.1155/2014/438531. The relative mRNA amounts of target genes were calculated after normalization to an endogenous reference gene (cytochrome C oxidase subunit 1, COX1). Results were expressed as relative amounts of mRNA compared to the vehicle control animals using the 2^−ΔΔCT^ formula [14].

### 2.6. Statistical Analysis

Statistical analysis of the data in this work was performed using two-way analysis of variance followed by pairwise comparison of selected means using the Student's *t*-test. The criterion for significance was set to *P < 0.05, **P < 0.01, and ***P < 0.001 as compared to the control.

## 3. Results 

### 3.1. Body Weight and Organ Wet Weights

**Figure 2:**
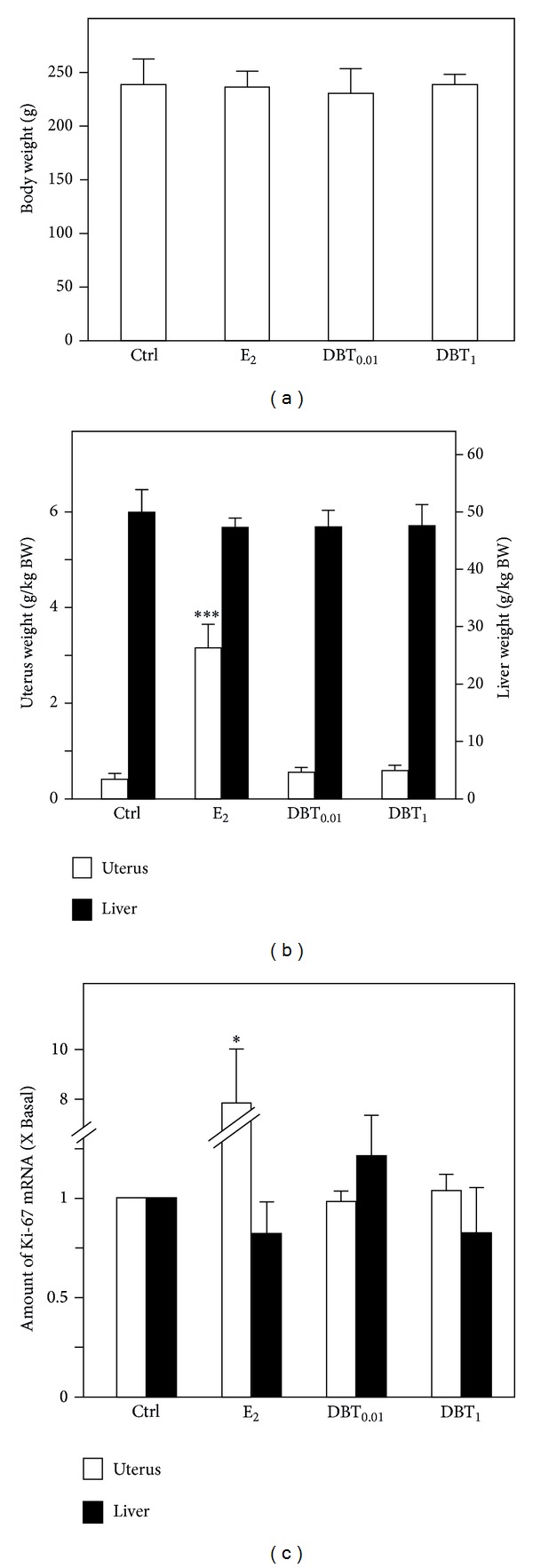
The effect of E_2_ or DBT on animal body and organ weight. (a) The ovariectomized animals were treated by E_2_ (at 1 *μ*g/kg BW/d; subcutaneous) or DBT (at 0.01 g/kg/d BW and 1 g/kg/d BW, orally) for three days; untreated ovariectomized animals served as a control group (Ctrl.). The body weight of rats before sacrifice was determined. The wet organs including uteri and livers were weighed. (c) The regulation of mRNA expression of the proliferation markers Ki-67 in uteri and livers was analyzed by semiquantitative real-time PCR analysis. Asterisks indicate values significantly different from the respective controls. *P < 0.05, **P < 0.01, and ***P < 0.001.

The uterotrophic response in ovariectomized rats was measured after 3 days of the drug treatment. DBT was applied at two doses of 0.01 (DBT_0.01_) and 1 (DBT_1_) g/kg BW/d. E_2_ was used as positive control at 1 *μ*g/kg BW/d, and the carrier castor oil was used as a negative control. These treatments could not impact on the body weight of animals (Figure 2(a)). Regarding the wet weights, E_2_ treatment for 3 days caused a significant increase in uterine wet weight of more than 5-fold but did not affect the liver weight (Figure 2(b)). The treatment with DBT, for both concentrations of DBT_0.01_ and DBT_1_, had no effect on the weight of uterus and liver (Figure 2(b)). This observation was in line with the expression of the mRNA of Ki-67, a sensitive proliferation marker in uterus, which however was unchanged after the DBT treatment (Figure 2(c)). The expression of Ki-67 mRNA is highly stimulated in response to the treatment with E_2_ (Figure 2(c)). In liver, none of the treatments resulted in a statistically significant impact on the expression of Ki-67 (Figure 2(c)).

### 3.2. Estrogen-Associated Gene Expression in Uterus and Liver

**Figure 3:**
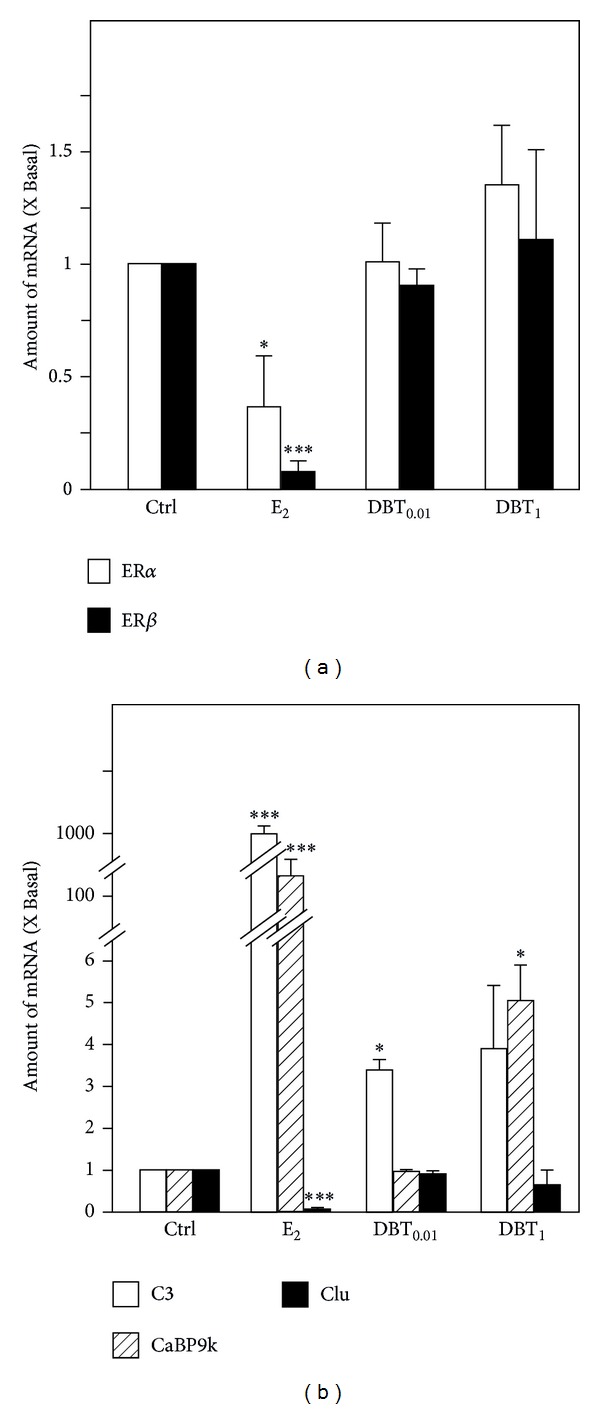
Regulation of expression of estrogen receptors and estrogen response genes in the uteri. (a) The mRNA expression of ER*α* and ER*β* in uteri and (b) the mRNA expression of estrogen response genes including C3, CaBP9k, and Clu were analyzed by quantitative real-time PCR analysis. Asterisks indicate values significantly different from the respective controls. *P < 0.05, **P < 0.01, and ***P < 0.001.

The mRNA expression of ER*α* and ER*β* had been detected in the rat uterus under all treatment conditions. After the treatment with E_2_, the ER*α* and ER*β* mRNAs showed the expected significant downregulation, as compared to the control animals, while DBT application could not affect the mRNA expression of ER*α* and ER*β* (Figure 3(a)). For ER action in the uterus, some very sensitive marker genes are known to monitor the estrogenic responses, that is, complement C3 (C3), calcium-binding protein 9 kDa (CaBP9k), and clusterin (Clu). In uterus, E_2_ treatment resulted in several hundredfold significant upregulation of the mRNA encoding C3 and CaBP9k, accompanied by a significant downregulation of Clu mRNA expression (Figure 3(b)). Apparently, DBT was capable of triggering very mild upregulation (not exceeding 5-fold) of the ER-dependent regulated genes C3 and CaBP9k. The higher dose of DBT caused a downregulation of Clu mRNA expression, which did not reach levels of statistical significance because of the high standard deviation of the mean value (Figure 3(b)).

**Figure 4:**
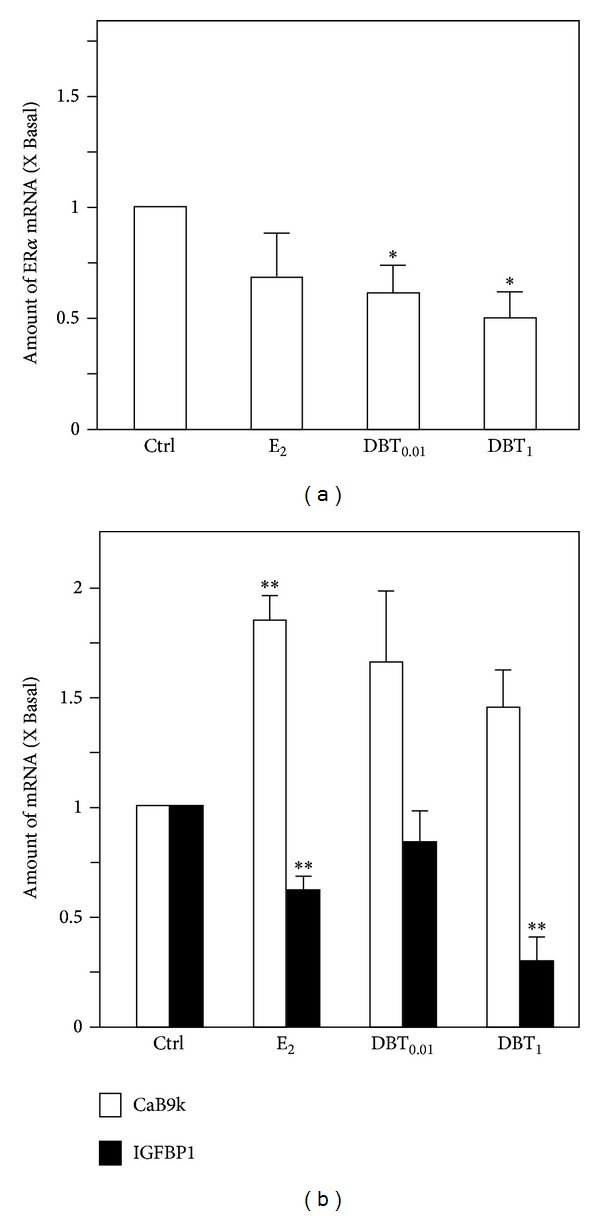
Regulation of expression of estrogen receptors and estrogen response genes in livers. The mRNA expression of ER*α* in livers (a) and the mRNA expression of the estrogen response genes including CaBP9k and IGFBP1 in livers (b) were analyzed by quantitative real-time PCR analysis. Asterisks indicate values significantly different from the respective controls. *P < 0.05, **P < 0.01, and ***P < 0.001.

The mRNA expression of ER*α* had been detected in the rat liver under all treatment conditions. The autoregulatory response of downregulation of ER*α* by E_2_ did not reach the level of statistical significance, whereas both DBT concentrations downregulated steady state mRNA levels of ER*α* in a statistically significant manner (Figure 4(a)). To assess the expression of ER*α*-dependent response genes in liver, the expression levels of suitable liver-specific ER response genes, that is, CaBP9k and insulin-like growth factor binding protein I (IGFBP1), were determined. The E_2_ treatment resulted in an upregulation of CaBP9k mRNA expression and in the downregulation of 1GFBP1 mRNA expression in the liver (Figure 4(b)). The treatment with high dose of DBT (DBT_1_) significantly reduced the expression of IGFBP1. The increased expression of CaB9k was revealed after DBT treatment at both concentrations but in both cases did not reach the level of statistical significance because of the variation (Figure 4(b)).

### 3.3. Lipid Metabolism-Associated Gene Expression in Liver

**Figure 5:**
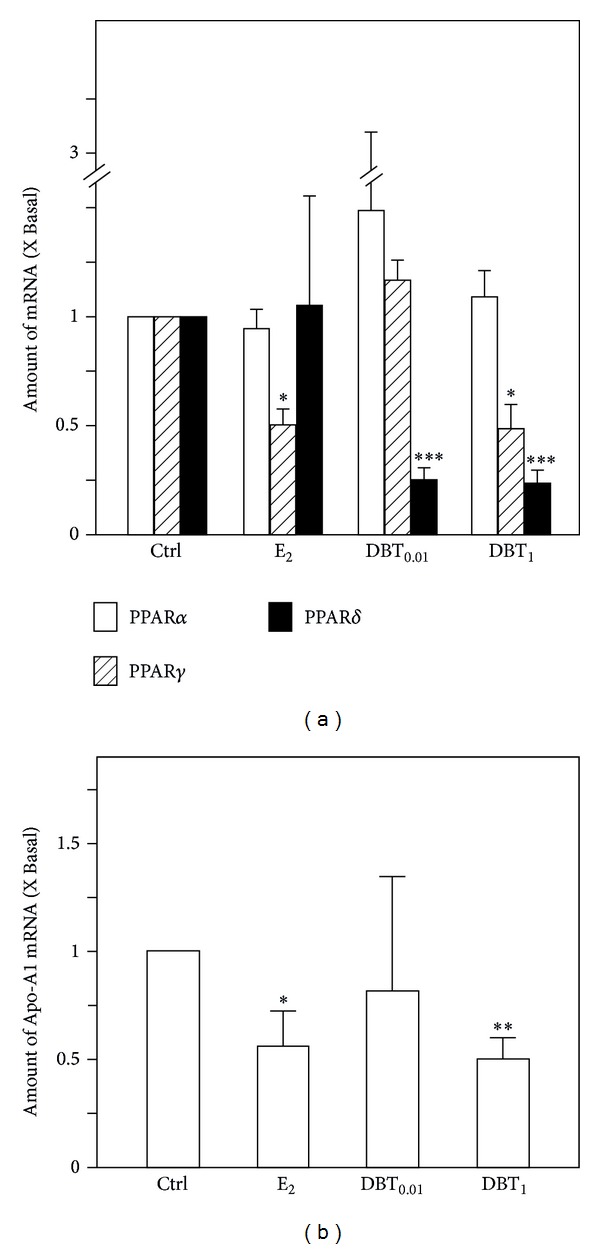
The gene expression of peroxisome proliferator-activated receptors and Apo-A1 in livers. (a) The mRNA expression of PPAR*α*, PPAR*γ*, and PPAR*δ* in livers was analyzed by quantitative real-time PCR analysis. (b) The mRNA expression of Apo-A1 in livers was analyzed by quantitative real-time PCR analysis. Asterisks indicate values significantly different from the respective controls. *P < 0.05, **P < 0.01, and ***P < 0.001.

Because DBT is believed to increase “Qi” we investigated the expression of genes associated with lipid metabolism. Being the major site for lipid metabolism, liver function is linked to obesity, which in turn is a risk factor for the diseases in hormone-dependent organs, like breast cancer. We therefore investigated the expression of Apolipoprotein A-I (Apo-A1) as the major component of HDL-cholesterol and of peroxisome proliferator-activated receptors (PPAR), which represent lipid sensors and are in various ways involved in the regulation of energy metabolism. The upregulation of PPAR*α* by DBT did not reach the level of statistical significance. Regarding PPAR*γ*, DBT at a concentration of 1 g/kg BW/d downregulated the expression of its mRNA (Figure 5(a)). This downregulation was similar to that induced by E_2_. On the contrary, an effect which was clearly independent of potential estrogenic properties of DBT was the pronounced downregulation of PPAR*δ* mRNA expression by both doses of DBT (Figure 5(a)). These results clearly point to the fact that DBT could regulate the expression of receptors intimately involved in the regulation of the availability and consumption of energy from fat through the modulation of expression of the receptors of the PPAR family. As a potential link to complications of the metabolic syndrome, regulation of cholesterol metabolism is of interest. We therefore assessed the regulation of expression of Apo-A1 mRNA. The expression pattern is indicative of an estrogen-like property of DBT in the concentration of 1 g/kg BW/d, which like E_2_ downregulated Apo-A1 mRNA expression.

### 3.4. The Regulation of DBT on Aryl Hydrocarbon Receptor Pathway in Uterus and Liver

Efficient detoxification is also crucial for health. Aryl hydrocarbon receptor (AHR) is known to trigger the expression of metabolizing/detoxifying enzymes in liver and other tissues, which is acting in a coordinated fashion with the battery of Nrf2-regulated enzymes. Here, we investigated the expression of AhR, AhR family members, and AhR responsive genes in uterus and liver by DBT in comparison to E_2_. Overall, we detected a tissue specific regulation of expression of AhR, AhR family members, and response genes following treatment with DBT.

**Figure 6:**
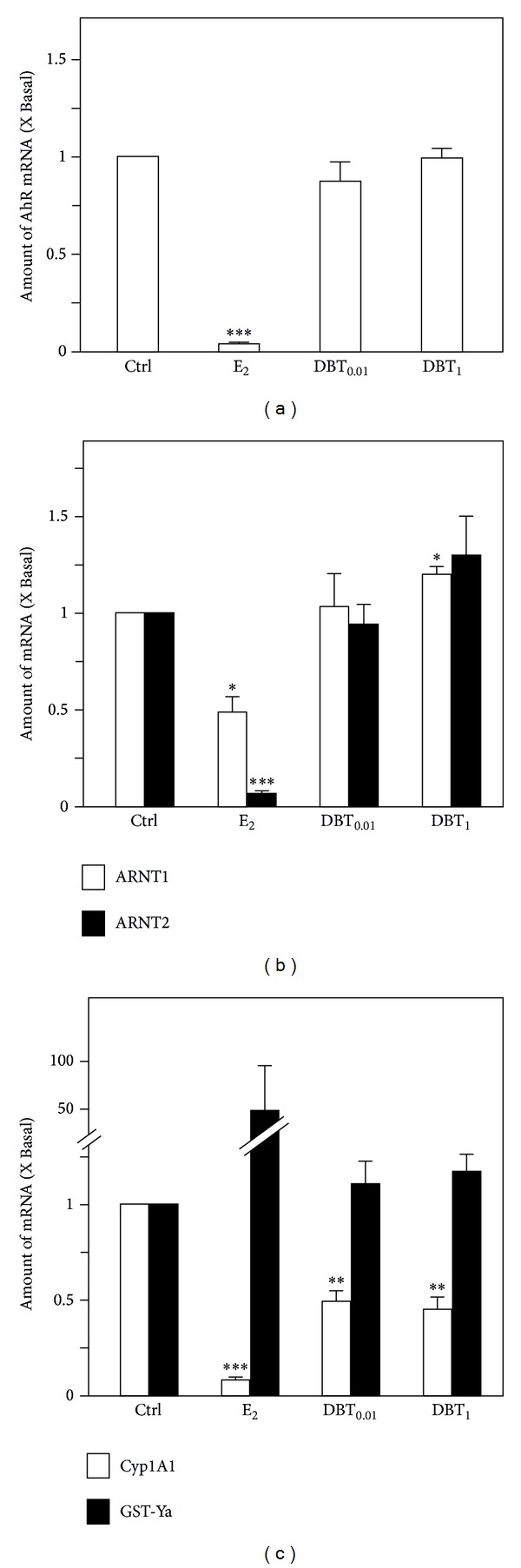
Regulation of expression of AhR-related genes in uteri. (a) The mRNA expression of AhR in uteri, (b) the mRNA expression of ARNT1 and ARNT2 in uteri, and (c) the mRNA expression of Cyp1A1 and GST-Ya in uteri were analyzed by semiquantitative real-time PCR analysis. Asterisks indicate values significantly different from the respective controls. *P < 0.05, **P < 0.01, and ***P < 0.001.

In uterus, AhR mRNA expression was strongly downregulated in response to E_2_, whereas no alteration was apparent in response to DBT (Figure 6(a)). The expression of aryl hydrocarbon receptor nuclear translocator 1 (ARNT 1) mRNA, the coregulator of AHR, was downregulated by E_2_, whereas DBT at the high dose leads to an upregulation of ARNT1 mRNA levels in this organ. Regarding ARNT2, a strong downregulation of mRNA expression resulted in the uterus in response to E_2_ treatment, an effect not detectable in response to DBT (Figure 6(b)). Cytochrome P450 (family 1) A1 (Cyp1A1) and Glutathione-S-transferases Ya (GST-Ya) are regarded as very sensitive response genes for AhR function. Cyp1A1 mRNA levels in the uterus were strongly downregulated by E_2_, an effect also detectable in response to both doses of DBT, but at a lower degree (Figure 6(c)). GST-Ya mRNA expression was only found to be upregulated in response to E_2_. DBT treatment in both doses did not affect GST-Ya mRNA expression in the uterus (Figure 6(c)).

**Figure 7:**
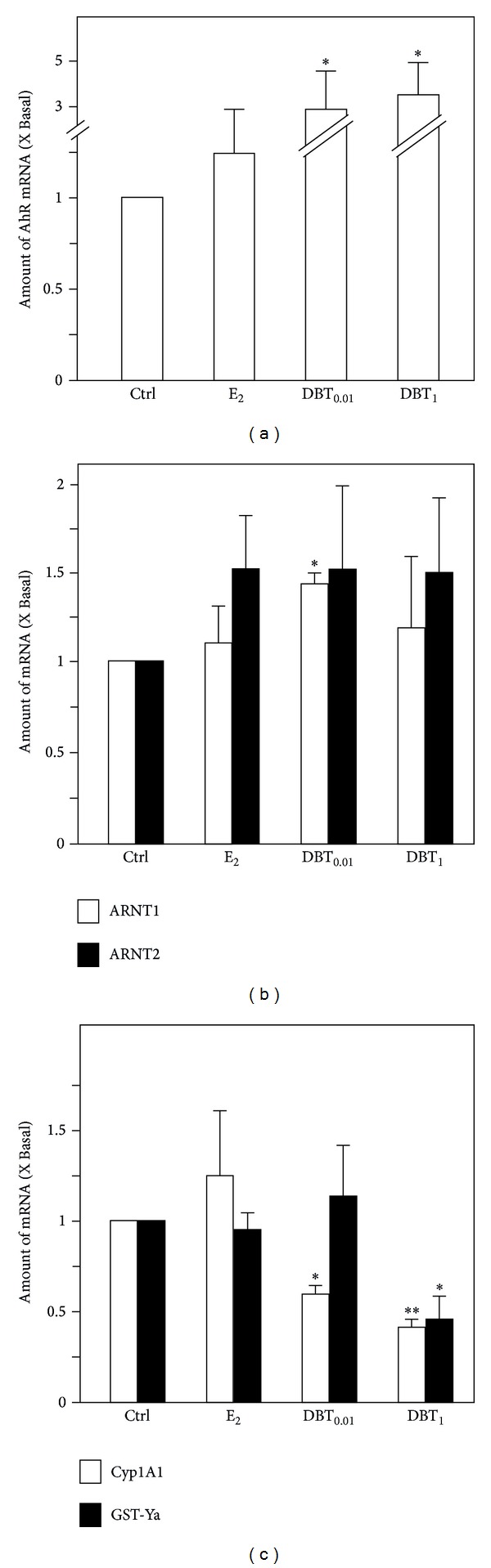
Regulation of expression of AhR-related genes in livers. (a) The mRNA expression of AhR in livers, (b) the mRNA expression of ARNT1 and ARNT2 in livers, and (c) the mRNA expression of Cyp1A1 and GST-Ya in livers were analyzed by semiquantitative real-time PCR analysis. Asterisks indicate values significantly different from the respective controls. *P < 0.05, **P < 0.01, and ***P < 0.001.

In liver, a mild, statistically not significant upregulation of AhR mRNA was detectable in response to E_2_ treatment, whereas DBT strongly induced AhR mRNA expression to over 3-fold (Figure 7(a)). The mRNA level of ARNT1 could only be induced by the low dose of DBT, but not for E_2_, and high dose of DBT. Furthermore, all three treatments appeared to upregulate ARNT2 mRNA expression; however, none of the values could reach the level of statistical significance (Figure 7(b)). E_2_ treatment resulted in a mild but not significant upregulation of Cyp1A1 mRNA levels, whereas DBT downregulates Cyp1A1 mRNA expression in a dose-dependent manner. On the contrary, E_2_ treatment did not result in an alteration of mRNA expression of GST-Ya, whereas DBT in high dose downregulated GST-Ya mRNA expression in a statistically significant, dose dependent manner (Figure 7(c)).

## 4. Discussion 

Though DBT exhibited potential estrogenic effects* in vitro *[5, 6, 8], the information on potential hormonal activities of DBT* in vivo* is limited. It is well known that proliferation of endometrial cells is under the control of estrogens and that the risk of endometrial carcinoma increases with estrogen replacement therapy [15]. In addition, since natural compounds with estrogen-like activities often exhibit organ selective properties* in vivo*, we evaluated the uterotrophic experiment performed in an organ dependent manner. We assessed potential effects of DBT in the uterus, as an organ of reproductive tract representing a classical target organ of estrogen action, and in the liver as the major metabolic site being one of the organs expressing the ER*α* predominantly. Therefore, to study the influence of DBT in the absence of endogenous estrogen, we used ovariectomized female rats to evaluate the ER selectivity of DBT in uterus and liver.

The results showed that DBT did not alter uterine and liver wet weight or the level of expression of proliferation markers and ERs at any of the investigated doses. For ER action in the uterus, some very sensitive marker genes are known which pick up estrogenic responses, that is, the upregulation of C3 and CaBP9k and the downregulation of Clu. C3 and CaBP9k could be upregulated by E_2_, whereas Clu was downregulated by response to E_2_ treatment in the uterus. DBT did very mildly mimic estrogenic responses for C3 and CaBP9k, but not for Clu. This is interesting as upregulation of C3 and CaBP9k involves ERE-response elements [16, 17] whereas the precise mechanism of ER mediated downregulation of Clu is not known. However, reflecting the transcription factor binding sites contained in Clu promoter involvement of SP1- and AP-mediated processes is likely [18]. In parallel, the liver changes of expression of CaBP9k and IGFBP1 had been established as estrogenic response markers, although the overall response was by far lower than the one detectable for CaBP9k or C3 in the uterus. In the liver, DBT again exerted weak estrogenic properties in the 3-day uterotrophic assay. Effects were only detectable using the most sensitive gene expression markers of estrogen action.

Liver in addition is a major site for lipid metabolism which is linked to obesity and which in turn is a risk factor for the diseases in hormone dependent organs like breast cancer. We therefore investigated the expression of Apo-A1 as the major component of HDL-cholesterol [19] and of PPAR receptors which are in various ways involved in the regulation of energy metabolism. They are also known targets for natural compounds and binding to them improves, for example, glucose uptake [20]. Our results clearly point to the fact that DBT through modulation of expression of the receptors of the PPAR family may contribute to the regulation of the availability and utilization of energy from fat. Numerous reviews summarize the regulation of metabolic pathways following activation of PPARs [21, 22], a topic which is out of focus of this paper. However, two features of our results regarding PPAR deserve discussion. A strong downregulation of PPAR*δ* in response to DBT was observed. In this connection it is important to mention that PPAR*δ* responded to DBT treatment apparently in an estrogen/ER independent manner. The functional consequences of downregulation of PPAR*δ* expression by DBT need to remain open at this point; however, it is not due to an autoregulatory downregulation of PPAR*δ* following binding of constituents of DBT, because DBT neither stimulated PPAR*δ* nor PPAR*γ*-dependent reporter gene activation in a transient transfection assay (data not shown). However, activation of a PPAR*δ* dependent signaling cascade by DBT would be interesting, as PPAR*δ* is the major regulator for mobilization of fat and energy expenditure from fat [22] and because of this phenotype potentially linked to obesity and its prevention.

In contrast, PPAR*γ* at least at a high dose of DBT responded in an estrogen-like manner by being downregulated. One important feature in addition to its insulin-sensitizing properties of PPAR*γ* is that it determines stem cell fate of mesenchymal stem cells. Skeletal effects of PPAR*γ* are well established in the meantime. It activates adipogenic differentiation, thereby inhibiting osteogenic differentiation [23, 24]. This situation would be disadvantageous for menopausal women and this is why downregulation of PPAR*γ* by DBT may point to a beneficial effect.

It is long known that the AhR pathway triggers the expression of metabolizing enzymes in the liver and in other tissues mostly in a coordinated fashion with the battery of Nrf2 regulated enzymes [25], thereby contributing to the detoxification process. We recently showed that there exists a link between estrogen function and regulation of AhR and members AhR signaling cascade in the uterus [26, 27]. Therefore, we comparatively investigated the regulation of expression of AhR, AhR family members, and AhR response genes in uterus and liver by DBT in comparison to estradiol. First, we confirmed the estrogenic response pattern of the AhR gene battery members [26]. For DBT treatment we overall detected a tissue specific regulation of expression of AhR, AhR family members, and response genes following treatment with DBT. Cyp1A1 and GST-Ya, in addition to being members of first (Cyp1A1) and second pass (GST-Ya) metabolic enzymes, are regarded as very sensitive response genes regarding crosstalk mechanisms of involving AhR and ER pathways [26] and here were tested too. As an interesting observation DBT treatment exhibited an estrogen-like response pattern for regulation of Cyp1A1 expression, but not for GST-Ya, indicative of the fact that DBT function in the liver is associated with both estrogen-like and estrogen-independent properties. The same holds presumably for AhR triggered responses. For the future it will be interesting to see whether DBT and/or its constituents will trigger AhR-mediated reporter gene activities.

In addition, although weakly, we found ARNT molecules to be upregulated by DBT in the liver. This is interesting as some of the authors involved in this paper recently described the upregulation of HIF1*α* by DBT ultimately leading to upregulation of erythropoietin [8]. ARNT1 is also called HIF1*β* and represents the heterodimeric dimerization partner of HIF1*α* in mediation of its nuclear responses [28]. In other words, we show here that DBT upregulates not only HIF1*α* but also HIF1*β*/ARNT1 thereby potentially supporting the effect of HIF1*α* on erythropoiesis.

In summary, we showed for the first time that DBT regulates the mRNA levels of members of the AhR signaling cascade, thereby exhibiting both estrogen-like and estrogen receptor independent activities, presumably leading to a distinct pattern of detoxification mechanisms. The final major result was that effects of DBT appear to be organ selective, influencing functions relevant to menopausal health. On the safety side there was no indication for estrogen dependent stimulation of proliferation within organs of the reproductive tract. Regarding efficacy DBT exhibits weak estrogenic properties and regulates the expression of functionally interconnected lipid sensors comprising amongst others the PPAR and AhR families of molecules [29, 30], the latter establishing a link to detoxification and energy metabolism. We hypothesize that DBT therefore may have properties which directly and indirectly impact on menopausal health.

## Supplementary Material

Gene expression analysis was performed in RNA preparations from tissue samples of uterus and liver. Information on analyzed genes, primer sequences used as well as amplicon sizes is given in Supplementary Table 1.

## Abbreviations

TCM: Traditional Chinese medicine
DBT: Danggui Buxue Tang
ASR: Angelicae Sinensis Radix
AR: Astragali Radix
AhR: Aryl hydrocarbon receptor
Apo-A1: Apolipoprotein A-1
E_2_: 17*β*-Estradiol
ER: Estrogen receptor
ERE: Estrogen response element
HRT: Hormone replacement therapy
PPAR: Peroxisome proliferator-activated receptor.
