# Supplementary material for: Functions of Danggui Buxue Tang, a Chinese Herbal Decoction Containing Astragali Radix and Angelicae Sinensis Radix, in Uterus and Liver are Both Estrogen Receptor-Dependent and -Independent
Source: Evid Based Complement Alternat Med. 2014 Aug 19;2014:438531. doi: 10.1155/2014/438531 (PMC4156991; doi:10.1155/2014/438531)
Supplement: Supplementary file 1 — Gene expression analysis was performed in RNA preparations from tissue samples of uterus and liver. Information on analyzed genes, primer sequences used as well as amplicon sizes is given in Supplementary Table 1. [file 438531.f1.pdf]

Supplementary Table 1 Primers used for real-time PCR quantification

Genes	Primer Sequences	Amplicon size (bp)
Proliferation-related Ki-67 antigen (Ki-67)	Forward 5' AAC CAG GAC TTT GTG CTC TGT AA 3'	209
	Reverse 5' CTC TTT TGG CTT CCA TTT CTT C 3'	
Estrogen receptor (ER) α	Forward 5' GGA AGC ACA AGC GTC AGA GAG AT 3'	383
	Reverse 5' AGA CCA GAC CAA TCA TCA GGA T 3'	
Estrogen receptor (ER) β	Forward 5' CTA CAG AGA TGG TCA AAA GTG GAA 3'	216
	Reverse 5' GGG CAA GGA GAC AGA AAG TAA GT 3'	
Complement C3 (C3)	Forward 5' ACA GCC TTC CCG GGA GCA TCA ACA 3'	276
	Reverse 5' AGC GCA CCA CAG GAG GCA CAG AGT C 3'	
Clusterin (Clu)	Forward 5' CCC TCC AGT CCA AGA TGC TCA ACA C 3'	303
	Reverse 5' CCA TGC GGC TTT TCC TGC GGT ATT C 3'	
Calbindin (CaBP9k)	Forward 5' TGT CTG ACT CTG GCA CTC ACT G 3'	181
	Reverse 5' CCT TCA GGA GGC TGG GGA ACT CTG 3'	
Insulin-like growth factor binding protein I (IGFBP1)	Forward 5' CAA CAG AAA GCA GGA GAT GAG A 3'	283
	Reverse 5' GAA GAA GGA GGG AGG AAA CAA C 3'	
Peroxisome proliferator-activated receptor (PPAR) α	Forward 5´ CTC TGG CCA AGA GAA TCC AC 3´	211
	Reverse 5´ CAG TGG AAG AAT CGG ACC TC 3´	
PPAR γ	Forward 5´ CAT TTC TGC TCC ACA CTA TGA A 3´	550
	Reverse 5´ CGG GAA GGA CTT TAT GTA TGA G 3´	
PPAR δ	Forward 5´ TCC AGA AGA AGA ACC GCA AC 3´	206
	Reverse 5´ GGC ATT GTA GAT GTG CTT GG 3´	
Apolipoprotein A-I (Apo-A1)	Forward 5´ CTG TGT ATG TGG ATG CAG TCA AG 3´	221
	Reverse 5´ GGT CCT TGT TCA TCT CGT TTC T 3´	
Aryl hydrocarbon receptor (AHR)	Forward 5´ CAA CCA AAC CAA ACA ACA GAG A 3´	196
	Reverse 5´ ATC CAT CAT CTT TCA ACC CAT C 3´	
Aryl hydrocarbon receptor nuclear translocator (ARNT) 1	Forward 5´ ACT GCT GCC TAC CCT ACT CTT C 3´	191
	Reverse 5´ GCT ACT TGG TTG TGC TGA TGT T 3´	
(ARNT) 2	Forward 5´ TGA AAG AAG GAG AAG CCC AAT A 3´	200
	Reverse 5´ CAT CAG AGT TAT GCC GAG ACA G 3´	
Cytochrome P450 (family 1) A1 (Cyp1A1)	Forward 5´ TTA TGA CCA CGA TGA CCA AGA G 3´	210
	Reverse 5´ GCC CTT CTA AAA TGT CCT GTA G 3´	
Glutathione-S-transferases Ya (GST-Ya)	Forward 5´ GCC AGC CTT CTG ACC TCT TT 3´	161
	Reverse 5´ GCT CCG CTA AAA CTT GAA AAT C 3´	
Cytochrome C oxidase I (COX1)	Forward 5' TGA GCA GGA ATA GTA GGG ACA GC 3'	261
	Reverse 5' GAG TAG AAA TGA TGG AGG AAG CA 3'	
